# Novel HIV-1 MiRNAs Stimulate TNFα Release in Human Macrophages via TLR8 Signaling Pathway

**DOI:** 10.1371/journal.pone.0106006

**Published:** 2014-09-05

**Authors:** Mark A. Bernard, Hui Zhao, Simon C. Yue, Asha Anandaiah, Henry Koziel, Souvenir D. Tachado

**Affiliations:** 1 Division of Pulmonary, Critical Care, and Sleep Medicine, Beth Israel Deaconess Medical Center and Harvard Medical School, Boston, Massachusetts, United States of America; 2 Department of Respiratory Medicine, The Second Hospital of Shanxi Medical University, Taiyuan, Shanxi, PR China; Helmholtz Zentrum Muenchen - German Research Center for Environmental Health, Germany

## Abstract

**Purpose:**

To determine whether HIV-1 produces microRNAs and elucidate whether these miRNAs can induce inflammatory response in macrophages (independent of the conventional miRNA function in RNA interference) leading to chronic immune activation.

**Methods:**

Using sensitive quantitative Real Time RT-PCR and sequencing, we detected novel HIV-derived miRNAs in the sera of HIV+ persons, and associated with exosomes. Release of TNFα by macrophages challenged with HIV miRNAs was measured by ELISA.

**Results:**

HIV infection of primary alveolar macrophages produced elevated levels of viral microRNAs vmiR88, vmiR99 and vmiR-TAR in cell extracts and in exosome preparations from conditioned medium. Furthermore, these miRNAs were also detected in exosome fraction of sera from HIV-infected persons. Importantly, vmiR88 and vmiR99 (but not vmiR-TAR) stimulated human macrophage TNFα release, which is dependent on macrophage TLR8 expression. These data support a potential role for HIV-derived vmiRNAs released from infected macrophages as contributing to chronic immune activation in HIV-infected persons, and may represent a novel therapeutic target to limit AIDS pathogenesis.

**Conclusion:**

Novel HIV vmiR88 and vmiR99 are present in the systemic circulation of HIV+ persons and could exhibit biological function (independent of gene silencing) as ligands for TLR8 signaling that promote macrophage TNFα release, and may contribute to chronic immune activation. Targeting novel HIV-derived miRNAs may represent a therapeutic strategy to limit chronic immune activation and AIDS progression.

## Introduction

Persons infected with HIV-1 exhibit a state of chronic immune activation, characterized by persistent and aberrant activation of immune cells, and increased tissue levels of pro-inflammatory mediators such as TNFα [1], that contributes to AIDS pathogenesis and may persist despite effective combined antiretroviral treatment (cART) [2]. The causes of HIV-induced chronic activation are not fully defined but likely include direct effects of viral proteins and nucleic acids, innate and adaptive immune responses to viral antigens, and translocation of microbial TLR ligands from the gut to the systemic circulation [1], [3], [4]. Chronic immune activation may play a role in the pathogenesis of AIDS, since natural hosts of simian immunodeficiency virus (SIV) such as sooty mangabeys fail to develop immunodeficiency and AIDS despite high levels of viral replication, while exhibiting surprisingly low levels of immune activation during the chronic stage of infection [5]. In contrast, SIV infection of rhesus macaques and other non-natural hosts results in high levels of systemic immune activation, CD4+ T-cell depletion and rapid progression to AIDS [6]. The absence of chronic immune activation in natural hosts during SIV infection supports the important role of chronic immune activation in AIDS pathogenesis.

MicroRNAs (miRNA; 18-22 nucleotide RNAs) are critical regulators of diverse cellular functions including proliferation, differentiation, metabolism, apoptosis and tumor progression through the canonical function of miRNA in targeted gene silencing by RNA interference (RNAi) [7]. However, miRNAs may also regulate cellular function independent of targeted gene silencing through stimulation of TLRs [8], [9]. Altered miRNA profiles are associated with progression or remission of inflammatory disorders such as rheumatoid arthritis, systemic lupus erythematosus and malignancies [10]. In addition, virus-encoded miRNAs can dysregulate host cell function, such as Epstein Barr virus (EBV) miRNA repression of host cell *CXCL11/ITAC*, inducing EBV-associated lymphomas [11]. Viral miRNAs from HIV have been described such as HIV vmiR-TAR [12]–[15], that may influence host cell function through RNAi function [16], but whether other biologically active HIV-derived miRNAs that can directly stimulate bystander or recipient host cells has not been established.

HIV-1 can infect macrophages, which may serve as a critical HIV reservoir [17]. Although macrophage infection is generally latent, activation can induce active replication from the HIV LTR with release of infectious virions [18], [19] and viral miRNA-TAR in exosomes [16]. In the current study focusing on human macrophages, we report on two novel HIV-derived miRNA (we denote as vmiR88 and vmiR99) that are released by HIV-infected macrophages and directly stimulate recipient macrophage early TNFα release that is dependent in part on macrophage Toll-like receptor 8 (TLR8). Furthermore, HIV-derived vmiRNA-mediated signaling in macrophages promoting TNFα release is dependent on high GU-content of HIV vmiRNA. Importantly, novel HIV vmiRNAs associated with exosomes are detected in sera of aviremic HIV-infected persons on stable cART. This is significant because even in suppressed viral replication, HIV miRNAs are produced since cART could not inhibit host RNA polymerase II from transcribing HIV mRNA in infected cells [20], [21]. Elevated levels of HIV miRNAs in the circulation may activate innate immune cells leading to immune activation and accelerate HIV-associated co-morbidities. Finally, antagomirs complementary to HIV-derived vmiRNAs dramatically reduce macrophage TNFα release. These data support a potential role for HIV-derived vmiRNAs from infected macrophages as contributing to chronic immune activation in HIV-infected persons, and may represent a novel therapeutic target to limit AIDS pathogenesis.

## Materials and Methods

### Reagents

HIV RNA oligoribonucleotide vmiR-TAR (Table 1) and novel HIV RNA oligonucleotides vmiR88 and vmiR99 (Table 1) and PCR primers were chemically synthesized (Integrated DNA Technologies, Coralville, Iowa). ssRNA40/Lyovec and ssRNA41/Lyovec were purchased from Imgenex (San Diego, CA). Lipid A, protease inhibitor mixture, phorbol myristic acid (PMA) and fetal calf serum were purchased from Sigma (St. Louis, MO). Cytokine ELISA kits were from R&D Systems (Minneapolis, MN). Oligonucleotides were complexed (50 µg/mL) in LyoVec according to the manufacturer's instructions (Imgenex, San Diego, CA) prior to treating cells.

**Table 1:** Oligoribonucleotides in quantitative Real Time RT-PCR and melting analysis of PCR products.

		qPCR product *T*_M_ (°C)	
ORN	Synthetic RNA sequence	Observed	Expected
vmiR88	5′-PO_4_-G*A*G*U*G*C*U*U*C*A*A*G*U*A*G*U*G*U*G*mU*mG-3′	70.5±0.2	71
vmiR99	5′-PO_4_-G*U*A*G*U*G*U*G*U*G*C*C*C*G*U*C*U*G*U*mU*mG-3′	70.2±0.3	70
vmiR-TAR	5′-PO_4_-C*U*A*A*C*U*A*G*G*G*A*A*C*C*C*A*C*U*mG*mC-3′	69.1±0.2	70
ssRNA40	5′-PO_4_-G*C*C*C*G*U*C*U*G*U*U*G*U*G*U*G*A*C*mU*mC-3′	70.5±0.4	71
antagomir88	5′-mC*mA*mC*mA*mC*mA*mC*mU*mA*mC*mU*mU*mG*mA*mA* mG*mC*mA* mC*mU*mC -3′	–	–
antagomir99	5′-mC*mA*mA*mC*mA*mG*mA*mC*mG*mG*mG*mC*mA*mC*mA*mC*mA*mC*mU*mA*mC-3′	–	–

“*”: phosphorothioate linkage

“m”: 2′-O-methyl modification

### Human macrophage cell lines

Human promonocytic THP-1 cells, promyelocytic cell line U937 (American Type Culture Collection), and HIV-infected U1 (HIV-infected U937 subclone) were obtained from the AIDS Research and Reference Reagent Program (Bethesda, MD). Cell lines were harvested during exponential growth phase, washed, and then incubated in complete medium (RPMI 1640 containing 10% heat-inactivated fetal calf serum, 2 mM glutamine, 100 units of penicillin, and 100 µg/mL streptomycin), differentiated with PMA (100 nM) for 24 h, adherent cells washed three times with complete medium, and then cultured in medium containing exosome-depleted FBS (System Biosciences, Mountain View, California). Macrophage differentiation was confirmed by CD11b expression and enhanced granularity by flow cytometry. Cells in complete medium were stimulated with oligoribonucleotide/LyoVec complexes or Lipid A (10 µg/mL) for 24 hr (37°C, 5% CO_2_), and conditioned medium was collected for TNFα analysis by ELISA.

### Human alveolar macrophages (AM)

Primary human AM cells were obtained from healthy 18–55 year old volunteers by bronchoalveolar lavage (BAL) using standard techniques [22]. All procedures were performed with written consent on adults following protocols approved by the Beth Israel Deaconess Medical Center Institutional Review Board and East Campus Committee on Clinical Investigations, New Procedures and New Forms of Therapy. Healthy subjects were without HIV risk factors and confirmed HIV seronegative by ELISA. BAL cells were separated from the pooled alveolar lavage fluid, and AM isolated by adherence [23]. AM viability was determined using trypan blue dye exclusion, and demonstrated >95% positive nonspecific esterase staining. AM in complete medium were stimulated with oligoribonucleotide/LyoVec complexes or Lipid A (10 µg/mL) for 24 hr (37°C, 5% CO_2_), and conditioned medium was collected for TNFα analysis by ELISA.

### Human sera

All procedures were performed with written consent on adults following protocols approved by the Beth Israel Deaconess Medical Center Institutional Review Board and East Campus Committee on Clinical Investigations, New Procedures and New Forms of Therapy. Archived sera from consenting asymptomatic HIV+ persons with peripheral CD4+ T-lymphocytes counts <200 cells/mm^3^ as detailed [24] were available for exosome preparation and HIV miRNA detection.

### ELISA

TNFα measurements of cell-free macrophage cultured supernatants were determined by ELISA (R&D Systems, Minneapolis, Minnesota) according to the manufacturer's instructions, and absorbance was measured at 450 nm using an Emax ELISA plate reader with multi-point data analysis using SoftMax Pro software (Molecular Devices, Sunnyvale, California). The detection limit for TNFα is 15.6 pg/mL. HIV-1 p24 antigen ELISA was from Zeptometrix (Franklin, Massachusetts). All measurements were performed in duplicate, and mean values of four measurements were used for statistical analysis.

### Time course analysis

THP-1 macrophages in complete medium were treated with vmiR99 (1.0 µg/mL) formulated in LyoVec at the indicated time points (37°C, 5% CO_2_). Conditioned medium was collected for TNFα analysis by ELISA. For analysis of cytokine mRNA, adherent cells were treated with Trizol (Applied Biosystems, Foster City, California) and total RNA was prepared according to the manufacturer's instructions. First strand cDNA was synthesized with the High Capacity RNA-to-cDNA kit (Applied Biosystems) using a GeneAmp PCR System 9600 (Perkin Elmer) set for 37°C for 60 min, 95°C for 5 min followed by 4°C. Real Time PCR was performed in 20 µL reactions with SYBR Select Master Mix (Applied Biosystems) using an ABI 7000 system programmed for 50°C (2 min), 95°C (2 min) followed by 40 cycles of 95°C (15 s) denaturation, 55°C (15 s) annealing and 72°C (1 min) extension. For cytokine expression analysis, qPCR of *TNF* cDNA was performed using forward primer 5′-TGCCTTGGCTCAGACATGTTT-3′ and reverse primer 5′-GCTACATGGGAACAGCCTATTGT-3′ [25]. For normalization, *GAPD* was detected using forward primer 5′- GGAGTCCACTGGCGTCTT-3′ and reverse primer 5′-AGGCTGTTGTCATACTTCTCAT-3′ [26]. Relative quantitation of *TNF* gene expression was calculated using the ΔΔ*C*_T_ method [27].

### TLR8 gene silencing in macrophages

To determine HIV miRNA-mediated signaling resulting in TNFα release by macrophages, targeted TLR8 gene silencing in human AM cells was performed as previously described [8].

### Exosome isolation

Exosomes were isolated from sera and cell culture conditioned medium using ExoQuick/ExoQuick-TC reagents (System Biosciences, Mountain View, California), according to the manufacturer's instructions. First flow-through fraction was analyzed for exosomal marker using anti-CD63 primary antibody, anti-rabbit HRP conjugate (System Biosciences) and ECL Select Western blotting detection (Amersham).

### RNA isolation

Total RNA was isolated from cells using the mirVana miRNA Isolation Kit (Life Technologies, Foster City, California) according to the manufacturer's instructions. Total RNA was extracted from exosomes using a SeraMir Exosome RNA Purification Column kit (System Biosciences) according to the manufacturer's instructions. Total RNA concentration was measured by absorbance (260 nm), and purity assessed by ratio of absorbance (260 nm and 280 nm) and agarose gel electrophoresis.

### Real Time RT-PCR analysis of miRNAs

First strand cDNA was synthesized from total RNA using a GeneAmp PCR System 9600 (Perkin Elmer), and PCR amplification was performed using the miRCURY LNA Universal RT microRNA PCR system (Exiqon Inc., Woburn, Massachusetts) on an ABI 7500 or ABI 7900HT Fast Real-Time PCR system (Applied Biosystems). Chemically synthesized microRNAs were used to standardize the assays in Absolute Quantitation mode. RNA samples were pre-diluted to 5 µg/mL in nuclease-free water. RNA is further diluted into a reverse transcriptase master mix using the provided reagents (Reaction Buffer, synthetic spike-in RNA UniSp6 and enzyme mix). The RT mixtures (20 µL/well) are incubated for 60 min at 42°C, 5 min at 95°C, and the cDNA products were cooled to 4°C. The miRCURY LNA Universal RT microRNA PCR system (Exiqon Inc.) was used for Real Time PCR analysis. Ten-fold serial dilutions of cDNA from synthetic microRNA were prepared in nuclease-free water as standards. cDNA from standards and samples were pre-diluted 80-fold into nuclease-free water containing ROX dye (50 nM for ABI 7500 instrument or 500 nM for ABI 7900HT; Applied Biosystems). The qPCR master mix was prepared from the provided reagents (SYBR Green master mix and PCR primers). Diluted standards and samples were combined with qPCR master mix and the plate was centrifuged (1500×*g* for 1 min at RT). The Real Time PCR instrument was configured for absolute quantitation of each amplicon. The instrument was set for 95°C for 10 min followed by 40 cycles of 95°C for 10 s and 60°C for 1 min (ramp rate = 1.6°C/s; 100% ramp rate in Standard mode) with detection of SYBR Green fluorescence. After cycling, melt curves were monitored to measure *T*_M_ of each PCR product, and absolute quantitation of microRNA from Real Time PCR was measured using Sequence Detection System software (Applied Biosystems).

### Cloning and sequencing of HIV miRNAs

PCR and cloning was used to confirm the sequences and ends of the candidate HIV miRNAs. Total RNA was isolated from HIV-infected cells and from exosomes and analyzed using miRCURY LNA Universal RT microRNA PCR (Exiqon, Woburn, MA). Selected qRT-PCR products were cloned into a pCR4-TOPO vector and chemically transformed into One Shot TOP10 chemically competent *Escherichia coli* (Applied Biosystems). Transformants bearing inserts were selected by spreading onto LB ampicillin plates, and individual clones were grown in LB ampicillin medium. Plasmids were purified using the PureLink Quick Plasmid Miniprep Kit (Applied Biosystems), annealed with M13(-21) forward primer (5′-TGTAAAACGACGGCCAGT-3′) or M13 reverse primer (5′-CAGGAAACAGCTATGAC-3′) followed by extension and chain termination with fluorescent dye-labeled dideoxy nucleotides. DNA sequencing in both directions was analyzed by capillary electrophoresis using an ABI 3730 DNA Analyzer (Dana Farber Molecular Biology Core facility, Boston, MA) and Sequence Scanner Software (Applied Biosystems).

### Statistical Analysis

Group comparisons were performed using two-way ANOVA using Prism 6.0 software (GraphPad Software, San Diego, CA) or one-way ANOVA with *post hoc* analysis by the Dunnett multiple comparisons test using InStat 3.0 statistical software (GraphPad Software, San Diego, CA). Results were expressed as mean±SEM. Statistical significance was accepted for *p*<0.05.

## Results

### Identification of putative candidate miRNAs encoded by HIV-1

**Figure 1:**
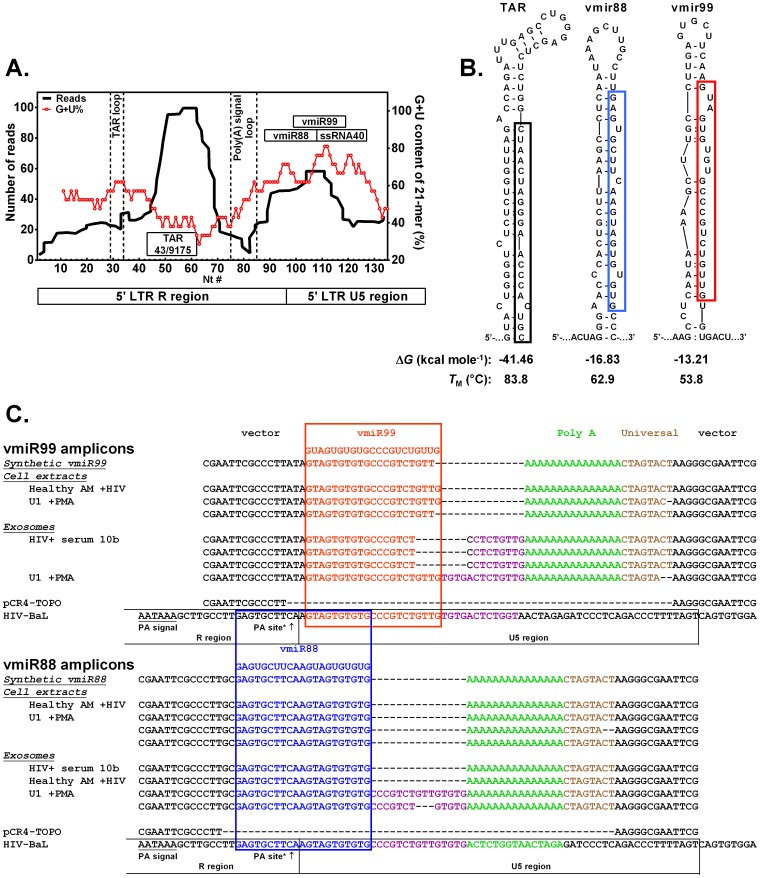
Identification of candidate miRNAs encoded by GU-rich tract in HIV LTR and is highly conserved in HIV-1. **A**: Small RNAs processed from HIV-1 LTR region observed by SOLiD Deep Sequencing. Left peak shows small RNAs derived from TAR stem (miR TAR). Right peak shows a hotspot for small RNAs derived from R and U5 stem region. The GU-rich tract (46 nt) encodes a family of viral miRs including vmiR88 and vmiR99. Modified from [14]. **B**: shRNA mirs are intermediates in biogenesis of mature vmiRs. shRNA reported for 43/9175 TAR (left). UNAFold software predicts folding of shRNAs vmir88 (middle) and vmir99 (right), which suggests the structures of intermediates in the biogenesis of the mature vmiR-TAR (black rectangle), vmiR88 (blue rectangle) and vmiR99 (red rectangle). UNAFold's thermodynamic calculations predict that all three shRNAs fold spontaneously (Δ*G*<0) into stable hairpins (high melting temperature, *T*_M_>53.8°C in 1M Na^+^). **C**: To delineate the boundaries and sequences of mature viral miRNAs, cell extracts and exosomal extracts were analyzed. Sample cell extracts were *in vitro*-infected AM (healthy AM+HIV), HIV-positive U1 macrophages stimulated by PMA (U1+PMA). Exosomal extracts were from exosomes of HIV+ human serum (HIV+ serum 10 b). Total RNA was amplified by qRT-PCR, cloned into pCR4-TOPO vector and DNA was sequenced. Sequences of vmiR88 and vmiR99 PCR products were aligned with sequences of plasmid (vector) and HIV-BaL strain. The polyadenylation signal (PA signal) and polyadenylation site (PA site) were reported [29], [36].

We previously reported that HIV ssRNA40 induced macrophage TNFα release via TLR8-mediated signaling and chromatin remodeling, and ssRNA40 biological activity was dependent on relative high guanosine + uridine (G+U) content [8]. Therefore, we sought to determine whether HIV produces other small non-coding RNAs such as miRNAs and ssRNA, which may be capable of activating innate immune cells such as macrophages. First, examining published Deep Sequencing data obtained from HIV-infected cells revealed peaks of short RNA reads throughout the HIV genome [14], and one of these peaks overlaps with the ssRNA40 sequence. We identified a GU-rich tract in the HIV LTR (Fig. 1A; nt#86-131 in R and U5 regions of HIV-1 BaL strain [Genbank: AB221005], that encompasses a hot-spot of short RNA reads indicative of possible mature microRNAs. We then used UNAfold RNA folding software [28] to identify RNA sequences featuring requisite short hairpin sequences (shRNA) in the HIV LTR region and to calculate hairpin stability (Fig. 1B). UNAfold identified three characteristic shRNA structures: vmir-TAR (Fig. 1B, left, mature miR highlighted in black) previously reported [14] in addition to two novel candidate shRNA, we denote as vmir88 (Fig. 1B, middle, mature form highlighted in blue) and vmir99 (Fig. 1B, right, mature form highlighted in red). Thermodynamic calculations of the change in Gibbs free energy for RNA folding (Δ*G*) were all less than zero (Fig. 1B) indicating that all three alternative RNA hairpins can form spontaneously (but mutually exclusively) in separate RNA molecules in the presence of 1 M sodium ion. The calculated melting temperatures (*T*_M_) for the three shRNA were all high (>53.8°C; Fig. 1B) and substantially above physiological temperature (37°C), predicting hairpin stability. Thus, using RNA folding analysis of published HIV Deep Sequence of HIV LTR [14], we identified vmiR-TAR (as previously reported) in addition to two novel mature viral miRNA candidates, vmiR88 and vmiR99. After reverse transcription using a degenerate primer with adapter and PCR amplification, DNA sequencing of PCR products of cDNA from synthetic vmiR88 shows full length sequence followed by the complement of the primer adapter (15-nt poly(A) and Universal Tag sequence) shown in Fig. 1C. Similarly, synthetic vmiR99 showed full length sequence (missing the final G) followed by a 15-nt poly(A) and Universal Tag sequence.

To validate and delineate the boundaries of mature viral miRNAs, cell extracts and exosomal extracts were analyzed. Sample cell extracts were *in vitro*-infected AM (healthy AM+HIV), HIV-positive U1 macrophages stimulated by PMA (U1+PMA). Exosomal extracts were from exosomes of HIV+ human serum (HIV+ serum 10 b). Analysis of RNA from HIV-infected U1 cells and *in vitro* infected AM cells revealed full-length vmiR88 and vmiR99 (Fig. 1C). One clone from U1 cells showed a missing 3′-terminal G like synthetic vmiR99. Furthermore, analysis of exosomal RNA exhibits full-length vmiR88 in clinical HIV+ serum of an asymptomatic person and in *in vitro* infected AM. However, exosomal miRNAs also demonstrated some longer variants. Exosomes from PMA-stimulated U1 cells produced vmiR88 with a 3′-terminal 15-nt HIV RNA extension (Fig. 1C) and vmiR99 with a 3′-terminal 13-nt HIV RNA extension (Fig. 1C). Analysis of vmiRs in exosomes from HIV+ serum of an asymptomatic person exhibited sequences from vmiR99 with four 3′ nucleotides substituted for 9 nt of HIV RNA (Fig. 1C). Observed sequencing of vmiR88 or vmiR99 have differing 3′ termini followed by polyadenylation that may have occurred *in vivo* and/or prior to first strand cDNA synthesis by *in vitro* polyadenylation. The 3′ termini of vmiR88 and vmiR99 lie downstream from the classical poly A site (Fig. 1C). Moreover, the observed vmiR88 sequence spans the classical poly(A) site [29], suggesting that a mechanism of alternative RNA folding and cleavage produces mature vmiR88. Interestingly, vmir88 is an extended shRNA hairpin structure compared to HIV-1 poly(A) hairpin, and the HIV-1 poly(A) hairpin was shown to regulate polyadenylation [30]. Observation of vmiR99 sequences from HIV-infected samples suggests a similar mechanism for vmiR99 biogenesis.

### Candidate miRNAs have high G+U base composition and are highly conserved in the HIV genome

**Figure 2:**
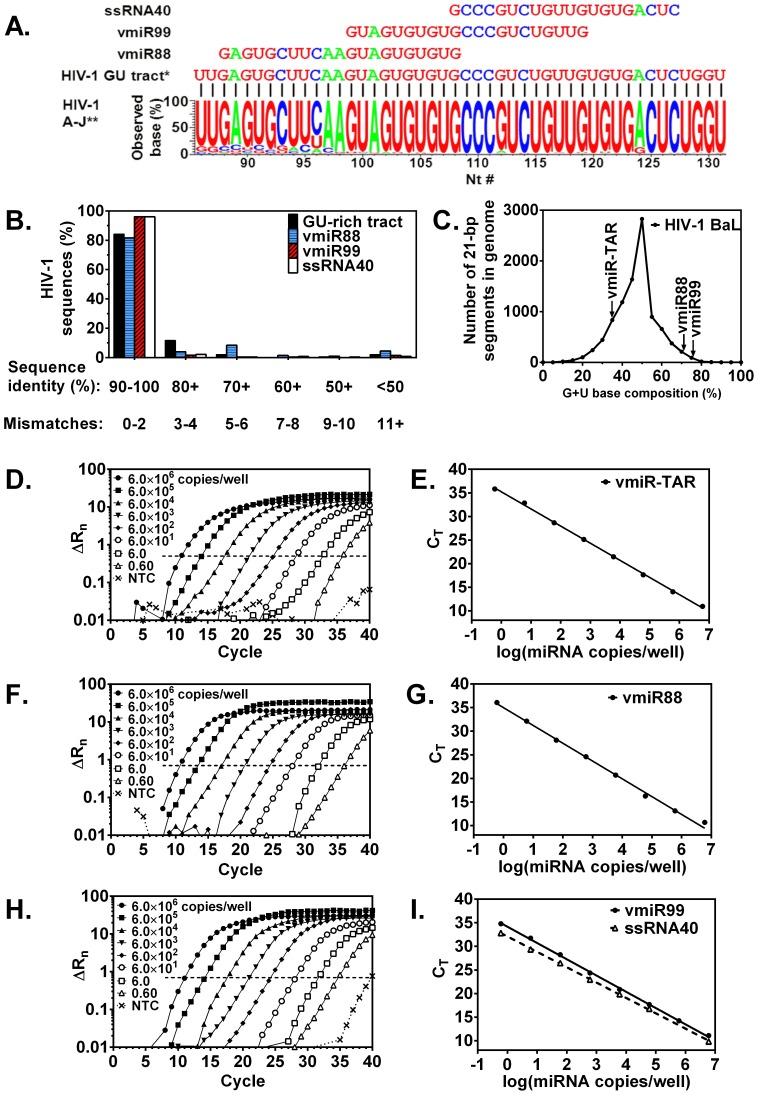
Sequence alignment of HIV vmiRs with consensus genomic sequence from HIV-1 subtypes and absolute quantitation of miRNAs by Real Time RT-PCR. **A**: Alignment of vmiR sequences of GU-rich tract is consistent with consensus genomic sequence from HIV-1 subtypes A-J, 533 isolates. **B**: VmiR99 is 90–100% identical to 96% of HIV-1 sequences. Sequences within the GU tract, vmiR88, vmiR99 and ssRNA40 were aligned with 196, 201, 254 and 272 genome sequences, respectively. **C**: Genomic RNA of HIV-1 BaL strain was scanned for every 21-bp RNA segment and the distribution of base compositions (46.5±11.8% G+U) is shown. VmiR-TAR is GU-poor (35%). VmiR88 and vmiR99 are GU-rich (71% and 76% G+U, respectively). Absolute quantitation of miRNAs was determined by Real Time RT-PCR. After first strand cDNA synthesis, amplification (**D, F, H**) and absolute quantitation (**E, G, I**) of vmiR-TAR (**D–E**) vmiR88 (**F–G**), vmiR99 (**H–I**) and RNA40 (I) was standardized using synthetic miR oligonucleotides in the miRCURY LNA Universal RT microRNA PCR method (Exiqon) on an ABI 7900HT FAST Real Time PCR system. ΔR_n_ is the change in normalized reporter fluorescence intensity. *C*_T_ is the threshold cycle in which the amplification curve crosses the dashed horizontal line. Data depict a representative experiment done in duplicate.

We recently demonstrated that high G+U content of ssRNA40 determined biological activity to stimulate macrophage TNFα release [8]. In addition, ssRNAs rich in G+U sequence have been shown to induce cytokines in plasmacytoid cells and peripheral blood mononuclear cells [31]. The potential importance of the G+U content is further suggested by high G+U sequence conservation despite high HIV mutation rate, based on analysis of the Los Alamos National Laboratories' HIV Sequence Database for HIV subtypes A-J [http://www.hiv.lanl.gov/content/sequence/HIV/mainpage.html]. The consensus sequence of the 46-nt GU-rich tract is strongly conserved with two or fewer mismatches in 84% of HIV genomic isolates (196 independent isolate sequences examined; Fig. 2A) of which 37% are completely identical to the consensus. Therefore, we next evaluated the GU content and sequence conservation of the candidate miRNAs. By scanning every miRNA-sized (21-bp) segment in the genomic RNA of HIV-1 _BaL_, we determined that these segments have base compositions of 46.5±11.8% G+U. TAR miRNA with only one UG is relatively GU-poor (35% G+U; Fig.2C). However, within the R and U5 regions, our two candidate mature miRs have very high G+U base compositions (vmiR88, 71% G+U and vmiR99, 76% G+U), which were over two standard deviations above average for HIV-1 _BaL_ strain (Fig. 2B). Moreover, the individual candidate miRNAs are highly conserved. VmiR99 is identical to 82% of genomic HIV sequences and has two or fewer mismatches in 96% of genomic sequences from 254 independent isolates (Fig. 2B). Similarly, vmiR88 is identical to 45% of genomic HIV sequences and has 0-2 mismatches in 82% of genomic sequences from 201 independent isolates (Fig. 2B). Thus, in addition to requisite hairpin structure, our novel mature miRNA candidates vmiR88 and vmiR99 were selected for biological investigation due to their relatively high G+U base composition.

### Sensitive Real Time qRT-PCR detects HIV miRNAs

Whether HIV miRNAs are released into biological specimens remains controversial, and may be in part be attributable to limitations in detection methodology. Recently reports describe HIV-produced miRNAs although at lower levels than both cellular miRNAs and miRNA produced by other viruses [32]. Deep Sequencing technology, which detects and sequences single RNA molecules, has recently identified numerous small RNAs of low abundance encoded throughout HIV genome including detection of HIV miRNA-TAR [14].

To address these limitations of detection, we developed a highly sensitive Real Time PCR method enhanced by Locked Nucleic Acid (LNA) primers for single-copy detection of HIV miRNAs. The assays were standardized using chemically synthesized miRNAs for absolute quantitation. Synthetic miRNA standards were reverse transcribed into cDNA, and Real Time PCR was performed using 10-fold serial dilutions of cDNA. This provided a concentration-dependent lag that precedes discernable exponential amplification as shown by monitoring normalized fluorescence intensity (Fig. 2D, F, H). The standard curves for vmiR-TAR, vmiR88, vmiR99 and ssRNA40 (Fig. 2E, G, I) demonstrate a seven-log analytical range including single-copy detection (log (copies/well) = 0). Thermal denaturation provided experimental melting temperatures (*T*_M_) of SYBR Green-bound PCR products in good agreement with the manufacturer's expected values (Table 1). Thus, sensitive LNA-enhanced Real Time PCR methodology offers a powerful approach to detect and analyze HIV-produced miRNAs of low abundance.

### Novel HIV-produced miRNAs detected in HIV-infected human macrophages

**Figure 3:**
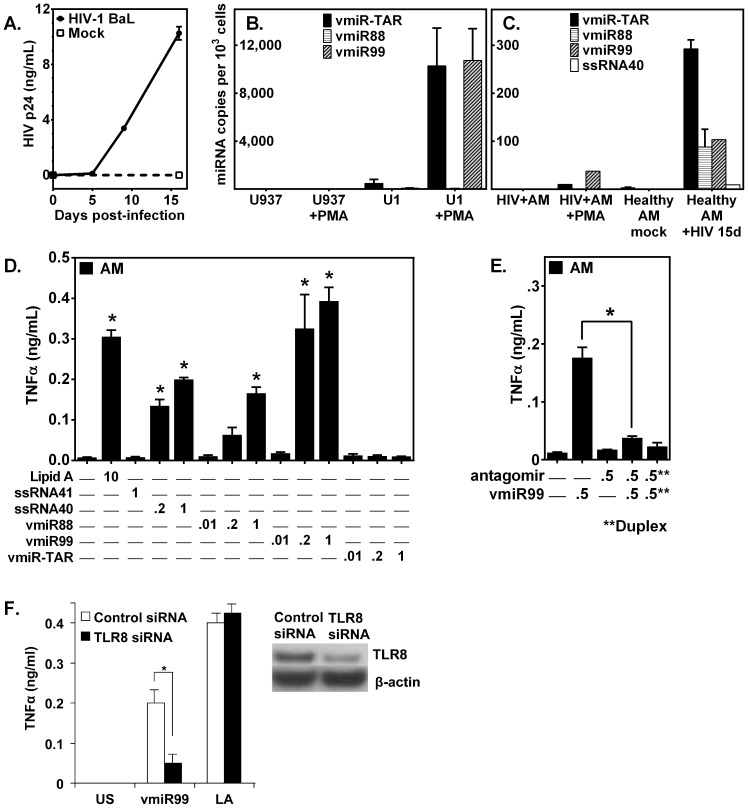
Novel HIV-produced miRNAs are detected in HIV-infected human macrophages, and stimulate macrophage TNFα release *in vitro*. **A**: AM were exposed to HIV-1 particles, BaL strain (10 ng/0.1 mL Gag p24/106 cells for 3 h) and washed. HIV p24 levels were assayed by ELISA. Data presented are AM infected with HIV-1 done in duplicate (*n* = 4 subjects). Quantitative PCR measurement of HIV miRNA from cell extracts of adherent (**B**) human macrophage cell lines U937 and HIV+U1, and (**C**) human primary alveolar macrophages (established *in vitro* HIV infection, or from asymptomatic HIV+ person), incubated in the absence or presence of PMA for 24 hr. **D**: TNFα measurement (ELISA) in culture supernatants in AM from healthy volunteers following 24 h incubation with HIV miRNA (vmiR-TAR, vmiR88, or vmir99) over a concentration range (0.01–1.0 µg/mL), lipid A (10 µg/mL), or control GU-rich ssRNA40 or AU-rich ssRNA41. **E**: TNFα measurement (ELISA) in culture supernatants from adherent human alveolar macrophages from healthy volunteers was treated with antagomir99 (1 h) followed by vmiR99 (24 h). At right, adherent AM were treated with pre-annealed antagomir99•vmiR99 duplex (**) for 24 h. **F**: TNFα measurement (ELISA) in culture supernatants from adherent human alveolar macrophages from healthy volunteers, in the presence of targeted TLR8 gene silencing (TLR8 siRNA) compared to control non-silencing RNAi (Control siRNA) following 24 h incubation with novel HIV vmiR99 (1.0 µg/mL in LyoVec), Lipid A (10 µg/mL) or unstimulated (US; LyoVec vehicle control). Cell extracts were analyzed by Western blot for TLR8 knockdown using anti-TLR8 antibody and for well loading using anti-β-actin antibody. Data for each figure reflect a minimum of 4 experiments, performed in duplicate. *, p<0.05.

Prior studies demonstrate release of HIV-produced vmiR-TAR by HIV-infected cells [12]–[15]. In the current study, vmiR-TAR was not detected in cell extracts of differentiated unstimulated HIV+U1 human macrophages, or the parent (non-HIV infected) U937 macrophages (Fig. 3B). However, following PMA stimulation, abundant HIV vmiR-TAR was detected in cell extracts from HIV+U1 macrophages. In addition, abundant novel HIV vmiR99 was detected in PMA-stimulated HIV+U1 macrophages, at levels comparable to vmiR-TAR (Fig. 3B). Using clinically relevant primary human alveolar macrophages, both HIV-produced vmiR-TAR and the novel vmiR99 were detected in cell extracts from alveolar macrophages from an asymptomatic HIV+ person, but only following PMA stimulation, and both were detected at relatively low copy numbers (Fig 3C). *In vitro* HIV infection of alveolar macrophages from healthy volunteers, demonstrates HIV p24 antigen at 16 d post infection, whereas mock infection produced detectable p24 antigen (Fig. 3A). Importantly, at day 16 following *in vitro* HIV infection, cell extracts yielded abundant expression of vmiR-TAR in addition to both novel HIV-produced vmiR88 and vmiR99, but not the control oligoribonucleotide, ssRNA40 (Fig. 3C). Thus, in addition to HIV-derived vmiR-TAR, HIV-infected macrophages produce abundant novel HIV-vmiR88 and vmiR99.

### Novel HIV-1 miRNAs stimulate macrophage TNFα release through TLR8 activation

HIV-produced vmiR-TAR can influence host cell function through canonical gene silencing in RNAi [33], but whether HIV-derived viral miRNA can directly stimulate macrophage responses has not been established. G+U-rich ssRNA40 has pro-inflammatory activity in macrophages [8]. Therefore, we tested whether vmiR88 and vmiR99 (viral miRNAs with high G+U content) can induce cytokine release. Human AM incubated with vmiR-TAR did not result in significant release of TNFα (Fig. 3D). In contrast, incubation with novel vmiR88 and vmiR99 resulted in robust release of TNFα in a concentration-dependent manner (Fig. 3D), with optimal TNFα release at 1 µg/mL. VmiR99 is notably more potent than vmiR88 in eliciting TNFα release in primary alveolar macrophages (Fig. 3D), although the uptake efficiency of LyoVec-vmiR complexes was not measured. This potency difference may be due to the relatively higher G+U content of vmiR99 (Table 1), as stimulation with a control molecule G+U-rich ssRNA40 also promoted macrophage TNFα release, whereas the inactive variant (GU-deficient ssRNA41) did not (Fig.3D). Importantly, on a molar basis, vmiR88 and vmiR99 stimulated TNFα release more potently than lipid A (Gram-negative bacteria-derived TLR4 ligand implicated in the gut translocation hypothesis of HIV chronic immune activation). VmiR99-mediated macrophage TNFα release was significantly inhibited in the presence of specific antagomir either pre-annealed to vmiR99 before addition to cells or by pre-treatment of macrophages with antagomir followed by vmiR99 challenge (Fig. 3E).

We recently demonstrated that HIV-derived ssRNA40 elicited TLR8-dependent release of TNFα [8]. To further study the mechanism of this pro-inflammatory function of HIV vmiRs, we investigated whether vmiRs also elicit TNFα response that is mediated by TLR8. Gene-targeted silencing of TLR8 confirmed that vmiR99-stimulated macrophage TNFα release is dependent in part on macrophage TLR8 expression (Fig. 3F). As expected, the TLR4 agonist Lipid A stimulated TNFα release without regard to knockdown of TLR8 (Fig. 3F). Western blot confirms that TLR8 protein was decreased in siTLR8-treated AM compared to non-silencing siRNA-treated AM, and uniform extract loading was demonstrated by anti-β-actin antibody (Fig. 3F). These data demonstrate that novel HIV-produced vmiR99 can directly stimulate signaling in macrophages that results in TNFα release and is dependent on macrophage endosomal TLR8.

### HIV vmiRNA-mediated macrophage TNFα release is rapid and inhibited by antagomirs

**Figure 4:**
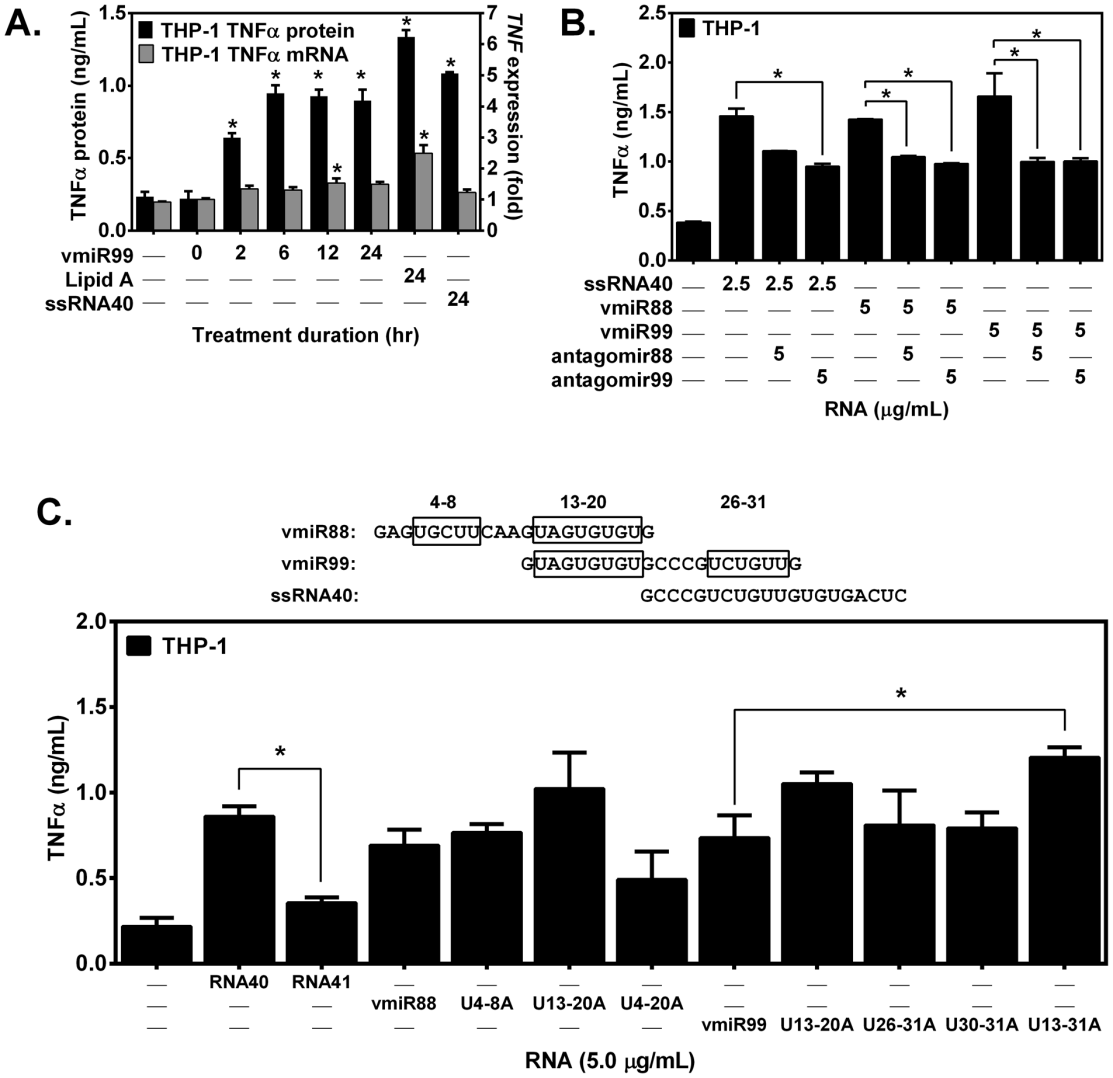
Viral miRNAs stimulate THP-1 macrophages to release TNFα rapidly in a vmiR sequence-dependent manner, and release is inhibited by antagomirs. **A**: THP-1 macrophages were treated with vmiR99 (1.0 µg/mL) at the indicated time points (hr) or with Lipid A or ssRNA40 (24 h). Conditioned medium was analyzed by ELISA. Total RNA was isolated from cell extracts, and expression of *TNF* (normalized by *GAPD*) was analyzed by qRT-PCR. Results are the average of three independent experiments done in duplicate. **B**: THP-1 macrophages were pre-treated with antagomir (5.0 µg/mL for 1 h) followed by treatment with ssRNA40 (2.5 µg/mL), vmiR88 (5.0 µg/mL) or vmiR99 (5.0 µg/mL for 24 h), and conditioned medium analyzed by ELISA. **C**: Sequence variants of vmiR88 and vmiR99 can elicit TNFα release by THP-1 macrophages. Variants of vmiR88 or vmiR99 were chemically synthesized by substituting the uridine residues of U-rich motifs (boxed regions) for adenine residues. VmiRs and variants (5.0 µg/mL) were applied to cells for 24 h. Supernatants of conditioned medium were assayed for TNFα by ELISA. *, p<0.05.

To determine the time course for HIV vmiRNA-mediated macrophage TNFα release, human THP-1 macrophages were incubated with vmiR99 and TNFα release measured in cell culture supernatants over 2–24 h. In response to vmiR99, half-maximal TNFα release was observed by 2 h with maximal release by 6 h, which remained stable through 24 h (Fig. 4A). By contrast vmiR99-induced gene expression was slow and transient; induction of *TNF* mRNA did not exhibit a statistically significant increase until 12 hr followed by a decline to basal expression by 24 h (Fig. 4A). Thus, vmiR99-mediated human macrophage TNFα release was rapid and consistent with release of pre-formed TNFα protein, suggesting direct stimulation rather than requiring vmiR99-targeted gene transcription, translation, post-translational modification, trafficking, externalization of TNFα protein or vmiR99-targeted silencing of genes regulating the pathway leading to activation of TNFα release.

Pre-treatment with antagomir (1 h) followed by vmiR treatment (perfectly complementary vmiR/antagomir pairs are strongly favored to form full-length 21-nt dsRNA duplexes (vmiR88/antagomir88 and vmiR99/antagomir99; Δ*G* = −31.0 and −38.4 kcal/mole, respectively) inhibited TNFα release (Fig. 4B). Surprisingly, half-complementary vmiR/antagomir pairs that can form half-length 10-nt dsRNA duplexes (vmiR88/antagomir99 and vmiR99/antagomir88; both with Δ*G* = −13.8 kcal/mole) were inhibited to the same extent as perfectly complementary pairs (Fig. 4B). Further, ssRNA40/antagomir88 have minimal complementarity (6-nt dsRNA duplex) but TNFα release is inhibited as well as perfectly complementary vmiR/antagomir pairs. Because an ssRNA40/antagomir88 duplex has poor thermodynamic stability (Δ*G* = −8.6 kcal/mole), inhibition by duplexing the signaling ligand (ssRNA40) can be ruled out in this case, suggesting an alternative mechanism of inhibition. These data suggest that targeted duplexing of vmiRNA with specific antagomirs is not essential to impairing vmiR88 or vmiR99-mediated TNFα release, but may require alternate mechanisms such as competitive binding to the ligand-binding site of cellular receptors such as TLR8.

### HIV vmiRNA-mediated macrophage TNFα release is dependent on vmiRNA sequence motifs

Small TLR8 agonists such as R848, CL075 and CL097 [34] are adenine (*A*) analogs, suggesting that adenine residues may be functional ligands of TLR8. However, ssRNA41 (an adenine-rich analogue of ssRNA40, with all *U* residues substituted with *A*) fails to stimulate TNFα release through TLR8 [8]. To further define the role of adenine in vmiR99-mediated signaling in macrophages resulting in TNFα release, we generated variants of vmiR88 and vmiR99 in which GU-rich motifs were mutated by substitution of U residues with A residues in selected blocks or along the entire vmiRNA (Fig. 4C). Macrophage TNFα release was minimal in unstimulated cells or in response to ssRNA41 (negative control), but robust in response to vmiR88, vmiR99 and ssRNA40 (positive control) stimulated TNFα release. Surprisingly, substitution of all U residues to A in vmiR99 (U13-31A) stimulated a statistically significant elevation of TNFα release compared to vmiR99 (Fig. 4C). Mutations of the middle block (U13-20A modification) of either vmiR88 or vmiR99 seemingly resulted in a modest increase in macrophage TNFα release, which was not statistically significant (Fig. 4C). Substitution of all U residues to A in vmiR88 (U4-20A) induced signaling that appeared to have decreased TNFα release compared to native vmiR88, but this was not statistically significant (Fig. 4C). These data demonstrate that specific nucleotide sequence and A-base composition, rather than simply A-content determines function of vmiR99. The uptake efficiency of LyoVec-vmiR complexes was not measured. The apparent potency of vmiRs to signal TNFα release by THP-1 macrophages (Fig. 4B, C) is lower than for AM (Fig. 3D), which could be due to different efficiencies of LyoVec-vmiR uptake in the two cell types.

### Novel HIV-produced miRNAs are released by infected macrophages and associated with exosomes

**Figure 5:**
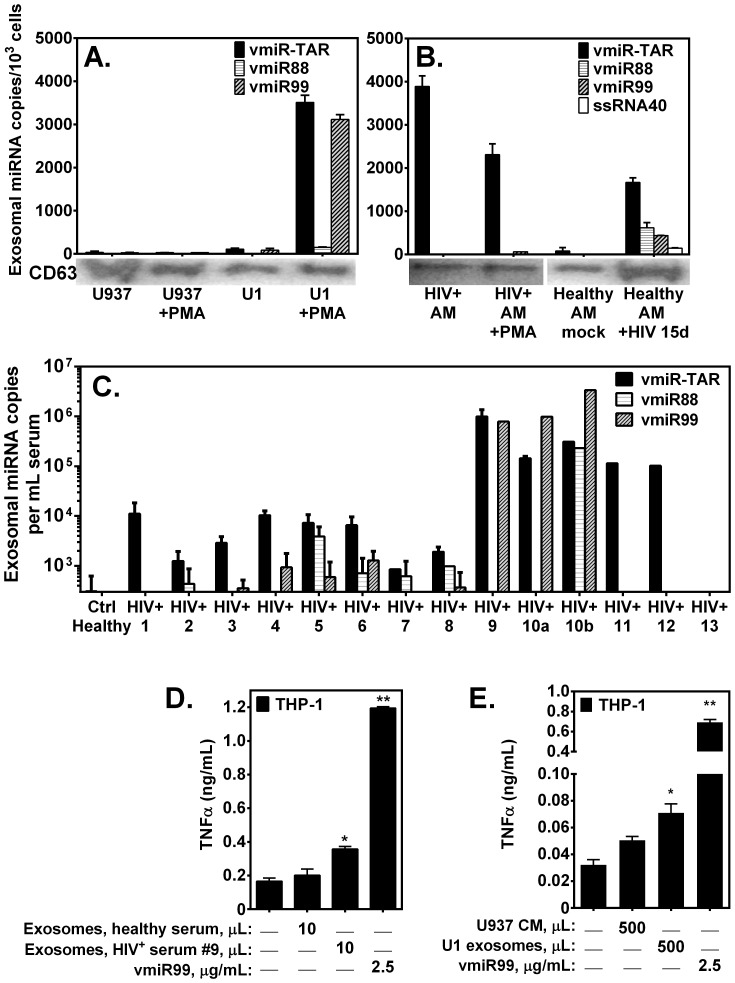
Novel HIV-produced miRNAs are released by HIV-infected human macrophages and associated with exosomes *in vitro*, and detected in sera from asymptomatic HIV+ persons. Quantitative PCR measurement of HIV miRNA from exosomal preparation of cultured supernatants from adherent (**A**) human macrophage cell lines U937 and HIV+U1, and (**B**) human alveolar macrophages (established *in vitro* HIV infection, or from asymptomatic HIV+ person), incubated in the absence or presence of PMA for 24 h. Western blot immediately beneath each bar graph demonstrates exosomal marker CD63 associated with corresponding sample. Data reflect a minimum of 4 experiments performed in duplicate. (**C**) Quantitative PCR measurement of HIV miRNA in exosomal preparations from archived sera of asymptomatic HIV+ persons with peripheral blood CD4+ T-lymphocyte count <200 cells/mm^3^. Data reflect measurements performed in duplicate. Results shown include exosome preparations isolated from HIV+ sera (*n* = 14) sampled from HIV+ patients (numbered 1–13). Serum samples “10a” and “10b” were drawn on separate days from Patient 10. (**D**) Exosomes were isolated from serum (HIV^+^ serum from Patient 9 or healthy serum) using ExoQuick-TC reagent and resuspended in the original volume of PBS (divalent cation-free). THP-1 macrophages cultured in medium (1.0 mL) were treated with 10 µL of exosome suspension (24 h, 37°C, 5% CO_2_). (**E**) THP-1 macrophages were treated (24 h) with 500 µL of conditioned medium (healthy parental U937 macrophages that had been cultured 6 d) or medium-suspended exosomes that had been isolated from 500 µL HIV^+^ conditioned medium (U1 macrophages cultured 6 d). Conditioned medium was analyzed by ELISA for TNFα. *, p<0.05.

A recent report demonstrates that HIV-derived vmiR-TAR released by HIV-infected cells was contained within exosomes [16]. Consistent with these observations, in the current study vmiR-TAR was detected in exosomal preparations from HIV+U1 human macrophages, but only following PMA stimulation (Fig. 5A). Importantly, HIV+U1 macrophages also released novel vmiR99, associated with exosomal fraction, at levels comparable to vmiR-TAR. There was no significant HIV-produced miRNA in the absence of PMA stimulation. In contrast, alveolar macrophages from HIV+ person exhibited robust constitutive release of vmiR-TAR associated with exosomes, but release of novel vmiR99 was limited, even following PMA stimulation. In comparison, *in vitro* HIV infection of primary alveolar macrophages from healthy persons resulted in release of vmiR-TAR and novel vmiR88 and vmiR99 in exosomal preparations (Fig. 5B). These data demonstrate that in addition to vmiR-TAR, HIV-infected macrophages release novel vmiR88 and vmiR99 associated with exosomes.

### Detection of novel HIV miRNA in exosome fraction of sera from HIV-infected persons

HIV TAR miRNA is associated with exosomes from the sera of HIV-infected persons [16], but whether other HIV vmiRNAs are present in sera has not been determined. In the current study, using sensitive quantitative RT-PCR, we detected HIV vmiR-TAR as well as novel vmiR88 and vmiR99 associated with the exosomal fraction of sera in the majority of HIV+ persons (Fig. 5C). The levels of vmiR88 and vmiR99 occasionally exceed those of vmiR-TAR, although there was extensive biological variability (Fig. 5C). Of 14 serum samples containing exosomes obtained from 13 HIV+ individuals at our clinic, at least one of these HIV miRNAs was detected from exosomal preparations from 12/13 (92%) of HIV+ individuals. Taken together, these data demonstrate that vmiR-TAR and novel HIV-produced miRNAs are present in exosomes from sera of HIV+ persons.

### Exosomes from aviremic HIV+ serum and from conditioned medium of HIV-infected cells stimulate TNFα release

Exosomes from HIV-infected serum and healthy serum were prepared using ExoQuick reagent. Serum exosomes from patient #9 were positive for vmiR99 and vmiR-TAR by qRT-PCR (Fig. 5C). Treatment of THP-1 macrophages with exosomes from healthy serum resulted in no stimulation of TNFα release compared to untreated macrophages (Fig. 5D). However, treatment by positive control (synthetic vmiR99) or by exosomes from HIV^+^ serum (patient #9) resulted in a significant increase in TNFα release (Fig. 5D). To confirm these results, we isolated exosomes from HIV-infected cell lines grown in medium containing exosome-depleted FBS (System Biosciences, Mountain View, CA). Exosomes isolated from U1 macrophages exhibit vmiR99 and vmiR-TAR by qRT-PCR (Fig. 5A). Conditioned medium collected from healthy parental U937 macrophages did not elicit additional TNFα release by THP-1 macrophages compared to untreated control (Fig. 5E). By contrast, synthetic vmiR99 and exosomes isolated from conditioned medium of U1 macrophages (each cell bearing two copies of integrated HIV-1) stimulated THP-1 macrophages to release TNFα (Fig. 5E). These results show that exosomes bearing viral miRNAs vmiR99 and vmiR-TAR can elicit TNFα release by macrophages.

## Discussion

These data demonstrate the production of HIV-derived miRNAs by human macrophages *in vitro*, including vmiR-TAR (vmiR43/9175) as previously reported [14], [33] in addition to novel HIV-derived vmiR88 and vmiR99. Novel HIV vmiR88 and vmiR99 are produced by HIV-infected human macrophage cell lines, human alveolar macrophages following *in vitro* HIV infection, and by alveolar macrophages from asymptomatic HIV-infected persons with advanced HIV infection (peripheral blood CD4+ T-lymphocyte count <200 cells/mm^3^), especially following PMA stimulation. Full-length sequences of vmiR88 and vmiR99 expressed in infected cells were confirmed. Exosome preparations harbored full-length vmiR88 as well as longer variants of vmiR88 and vmiR99 bearing 3′ extensions of viral sequence. Furthermore, incubation of uninfected recipient macrophages with exogenous vmiR88 or vmiR99 stimulate a pathway in macrophages that elicits TNFα release. The mechanism of these pro-inflammatory miRNAs was not due to the role of miRNA in targeted gene silencing by RNA interference. Instead, the HIV-derived miRNAs directly stimulated a signaling pathway in macrophages resulting in TNFα release, a process that was dependent partly on G+U base composition of the miRNA, and partly on macrophage TLR8 expression. Using a flow cytometry based fluorescence resonance energy transfer (FC-FRET) assay, we demonstrated binding of ssRNA40 to human TLR8 [35]. Furthermore, TNFα release was inhibited by antagomir88 and antagomir99 even with partial or little complementarity to the ssRNA ligand, suggesting that these antagomirs may function more strongly as receptor antagonists relative to their intended function as ligand antagonists. Finally, novel HIV vmiRNAs are detected in sera of HIV-infected persons, and associated with exosomal fraction. Biological significance is suggested by the finding that exosomes from serum of an HIV-infected aviremic person as well as exosomes from HIV-infected U1 macrophages elicit a pro-inflammatory response (TNFα release) by human macrophages, whereas exosomes from healthy serum and from uninfected parental macrophages did not stimulate TNFα release. These data support a potential role for novel HIV-derived vmiRNAs from macrophages as contributing to chronic immune activation in HIV-infected persons.

This is the first study to detect novel HIV vmiR88 and vmiR99 in biological samples, and their association with exosomes from human macrophages *in vitro* and the clinical relevance of vmiR88 and vmiR99 through detection in exosomal preparations from the sera of HIV+ persons, which suggests the possibility of exosome-mediated delivery of pro-inflammatory viral miRNAs to uninfected bystander cells. Highly sensitive detection was possible using an advanced qRT-PCR methodology enhanced with LNA primer technology. Sequences of qRT-PCR products of vmiR88 and vmiR99 from cell extracts were confirmed and exhibited 3′ termini that are distinct from the reported 3′-terminal polyadenylation site of HIV genomic RNA [29], [36]. Longer variants of vmiR88 and vmiR99 were detected in exosome preparations, which may be related to selective miRNA packaging into exosomes or have unknown functions.

The mechanism for novel vmiR88- and vmiR99-induced macrophage TNFα release was dependent in part on high G+U base compositions of the miRNA, as vmiR-TAR (35% G+U) and control ssRNA41 (absent G) failed to stimulate signaling resulting in macrophage TNFα release, whereas vmiR88 (71% G+U), vimR99 (76% G+U) and positive control ssRNA40 (65% G+U) stimulated macrophage TNFα release, although the minimal requirement was not established in the current study. For comparison, genomic RNA of HIV-1 BaL strain has 47% G+U composition. Importantly, the observation that the molar potency of vmiR99 to induce macrophage TNFα exceeded that for the endotoxin component lipid A by 40-fold, suggesting that even low concentrations of vmiR88 and vmiR99 may significantly contribute to signaling in macrophages that result in downstream pro-inflammatory cytokine release.

Although vmiR88 and vmiR99 exhibit sequence overlap, each demonstrates distinct function. In the current study each novel vmiRNA induces macrophage signaling culminating in TNFα release, but vmiR99 more potently than vmiR88. Also, U→A mutations of vmiR99 modifies the potency for macrophage TNFα release; substitution of every U to A along the entire vmiR99 sequence enhances macrophage TNFα release, which suggests that G+U content may contribute to stimulation of macrophages resulting in TNFα release, but other molecular sequences may be more important. Indeed TLR8 is stimulated by ssRNA molecules of many different sequences with various potencies [31]. Prior studies indicate the importance of nucleotide sequence, although secondary structure may be more critical to RNA function, with the poly(A) hairpin [29], [36] providing essential stability [30], [37]. In addition, the striking observation that both vmiR88 and vmiR99 were identified from a highly conserved GU tract in a virus with an exceptionally high mutation rate suggests critical, perhaps essential, functions for these HIV-derived miRNAs. Although vmiR-TAR may modulate cellular apoptosis, additional roles of novel vmiR88 and vmiR99 remain to be determined. RNAs can fold into different secondary structures during transcription, which may explain how miRNA biogenesis can yield distinct vmiR88 and vmiR99 products of overlapping primary sequence by processing alternatively-folded transcripts. This idea is precedented by bacterial attenuators, which are the microbial RNA sequences that form alternative, mutually exclusive hairpin loops (terminator hairpins or anti-terminator hairpins) to regulate expression via transcriptional pausing mechanism according to physiological conditions [38]–[40]. Similarly, it was recently reported that during transcription of the HIV LTR, two host enzymes of the RNAi pathway (DROSHA, DGCR8) and other factors cause pausing of RNA polymerase II and endonucleolytic cleavage in the TAR hairpin [41], but that study did not include the immediately downstream sequences (vmir88 and vmir99, alternative hairpins of comparable thermodynamic stability) and could not study vmiR88 and vmiR99 miRNA biogenesis.

Results from the current study support an important biological function for these novel HIV-derived miRNAs other than RNAi function. In general, miRNA function is characterized as RNA interference through targeted gene silencing of mRNAs at the translational level [42], [43]. However, the observed macrophage response to vmiR88 and vmiR99 was much faster than would be expected for miRNA RNAi function, and rather supports other important biological miRNA function(s), *eg*. serving as direct agonists for cell signaling, such as ligands of TLR8. VmiR99-stimulated TNFα release was very rapid with >50% released by 2 h and maximal release by 6 h, which is much faster than the 12 h for induction of *TNF* gene expression. The rapid time course is consistent with release of pre-formed TNFα protein rather than reflecting *de novo* cytokine synthesis, post-translational modification, trafficking and externalization, suggesting the predominant influence of vmiR88 and vmiR99 was independent of gene silencing pathways. Additional support for non-RNAi function of the novel vmiR88 and vmiR99 relates to their copy numbers, as host cell miRNAs generally far exceed small RNA reads in HIV-infected cells [14], [15], [32]. In general, the RNAi function of miRNA is stoichiometrically dependent, since RNAi translational blockade requires ≥100 miRNA copies for effective silencing of individual genes by the mechanism of RNAi [44]. However, as HIV-derived vmiR88 and vmiR99 can be released at low copy numbers, the observed biological effects more likely represent activation of alternate pathways (such as TLR8 signaling) that can provide amplification in a signal cascade and stimulate physiologically relevant responses resulting in cytokine release.

Detection of abundant vmiR-TAR in HIV-infected cells associated with exosomes in the current study confirms reports by other investigators [12], [45], and validates our methodology. Although abundant, the observation that vmiR-TAR did not stimulate macrophage TNFα release suggests different regulatory roles for HIV-derived miRNA. VmiR-TAR may influence cellular apoptosis or enhance macrophage susceptibility to HIV infection [16], [46] through targeted gene silencing, although this was not specifically investigated in the current study. Though the current study focused on novel HIV vmiR88 and vmiR99, the potential identification and role of other pro-inflammatory HIV-produced miRNAs cannot be excluded and remains the focus of active investigation. Furthermore, the potential role of other HIV-produced miRNAs that may serve an antagonistic or anti-inflammatory role cannot be excluded, and any RNAi influence of novel vmiR88 or vmiR99 cannot be excluded, as these were not specifically investigated in the current study. The potential influence of contaminating HIV virions on macrophage release of TNFα cannot be excluded, as the exosome preparations were not specifically processed to remove HIV-1 virions [47]. However, experiments using synthesized vmiR88 and vmiR99 suggest that these molecules are sufficient for signaling resulting in induction of macrophage TNFα release (in the absence of HIV-1 virions). Moreover, abundant vmiRs were measured in exosomes from sera of asymptomatic HIV+ persons even though they exhibit clinically undetectable viral loads. We are investigating whether vmiR uptake is more efficient depending on vehicle (exosome delivery compared to synthetic LyoVec complexes) or cell type, since uptake efficiency would affect the apparent dose of vmiR needed for proinflammatory cell signaling leading to cytokine release. Although novel HIV-produced vmiR88 and vmiR99 promote macrophage TNFα release *in vitro* cell assays (current study) and stimulates foam cell formation *in vitro* [35], whether this reflects biological activity *in vivo* remains to be determined. We have previously demonstrated that HIV ssRNA stimulates macrophages via TLR8 (and not by TLR7) resulting in TNFα release [8]. We report that HIV vmiR99 also stimulates the same response via TLR8 in gene silencing experiments. Because TLR7 was not specifically investigated in the current study, we cannot exclude the possibility that vmiR99 may also interact with macrophage TLR7 (or other TLRs). Finally, in addition to the intended vmiR-specific blocking activity of antagomirs, our antagomir designs were fully 2′-O-methylated and might also serve as TLR8 antagonists, since in human PBMCs the related receptor TLR7 is inhibited by direct binding to alternating 2′-O-methylated ssRNA and dsRNAs [48],[49].

## Conclusions

**Figure 6:**
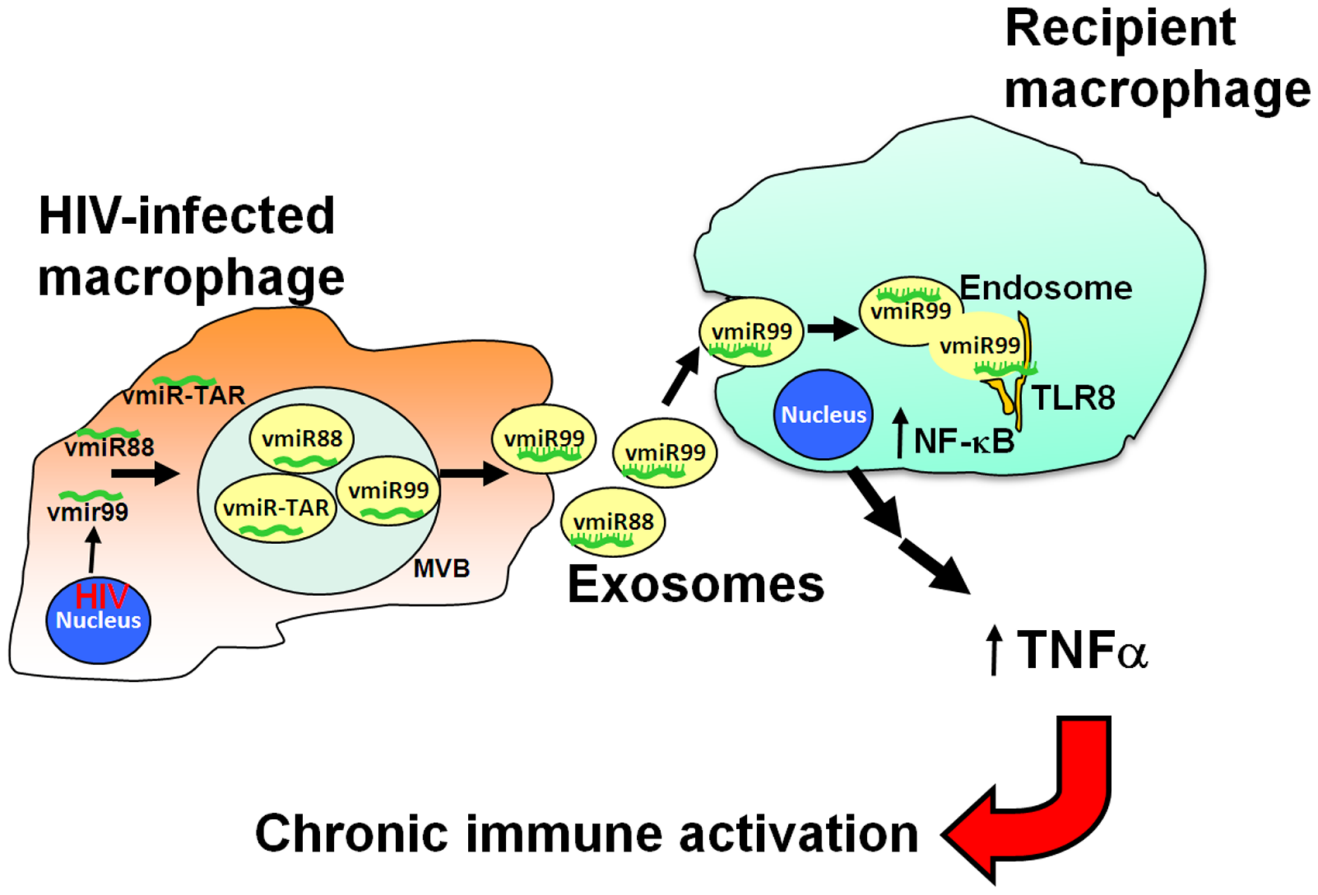
Working model of HIV-produced miRNA activation of bystander cells in HIV+ persons. This figure provides a working model describing how HIV-produced miRNAs are encapsulated in exosomes and released from HIV-infected macrophages into the circulation. Host RNA polymerases transcribe HIV genomic RNA, which is then exported into the cytoplasm and packaged into infectious virions. However, some HIV transcripts can be processed in the host RNA interference pathway into mature viral miRNA. HIV miRNAs are packaged into multi-vesicular bodies and released by macrophages encapsulated in exosomes. Exosomes are disseminated either locally or systemically to be taken up by bystander macrophages, trafficking GU-rich vmiR88 and vmiR99 to the endosomal TLR8. Through this non-RNAi function of miRNAs that is distinct from the well-established role of miRNAs in RNA interference, vmiR88 and vmiR99 induce TLR8-mediated inflammatory signaling pathway that leads to downstream release of TNFα leading to chronic immune activation.

This study demonstrates that HIV encoded miRNA, including vmiRNA-TAR and two novel HIV miRNA, vmiR88 and vmiR99, are detected in HIV-infected human macrophages, are released by HIV-infected macrophages and are associated with exosomes. Furthermore, mature vmiR88 and vmiR99 demonstrate an important biological function other than RNAi function and can directly stimulate signaling in macrophages that elicits TNFα release, dependent on TLR8 and G+U content. The detection of HIV vmiR88 and vmiR99 in the exosomal fraction of sera from HIV+ persons and pro-inflammatory stimulation by vmiR-associated exosomes raises the possibility that circulating vmiR88 or vmiR99 can stimulate recipient macrophages *in vivo*, and together with other circulating microbial TLR ligands such as endotoxin may contribute to chronic immune activation (Fig. 6). Furthermore, specifically targeting HIV-produced vmiR88 or vmiR99 with molecules such as antagomirs may represent a novel therapeutic strategy to limit chronic immune activation and the progression of AIDS. HIV miRNAs may also serve as biomarkers for future development as clinical diagnostics.
